# Progress in Surgery

**Published:** 1899-04-22

**Authors:** 


### Progress in Surgery.

### GENITO-URINARY SURGERY.

(*Continued from page* 436, *Vol. XXV.*)

**Prostatic Hypertrophy** is at all times an anxious condition to treat, and in the absence of rules of action we must be guided by individual experiences. Much attention has been devoted to this subject in America. Eugene Fuller calls attention to the variations in the character and form of the hypertrophy,[1] no two cases being quite alike. He thinks that in certain cases restoration of the function of the bladder may be restored, even though complete atomy may have existed. Operation in the aged should be expeditious. Fuller favours a modification of McGill's suprapubic prostatectomy by incising the posterior part of the obstructing mass by serrated scissors, and then enucleating the prostatic tissue. He then makes an opening in the perineum, through which he passes a tube for drainage, and sews up the suprapubic wound completely, except for a small space for drainage, where a temporary suture is placed. In some cases the finger has not sufficed for enucleation, but the use of strong forceps has been requisite. In sewing the bladder he has now discarded separate catgut sutures, and sews the parietes and bladder in one. Three out of five of the patients with marked atheroma of the arteries died. He summarises his opinion in the words, "there is no class of results the contemplation of which gives me greater pleasure than those following prostatectomy."

Dr. Willy Meyer gives his experience of Bottini's operation.[2] He regards the effect of the instrument in dividing the sphincter vesicæ muscle as comparable to the dilatation of the sphincter ani in setting the part at rest. He records unexpectedly good results from twelve cases, with a mortality of about 8 per cent. Bottini and Freudenburg have been the pioneers in this operation, with some ninety cases between them. Willy Meyer has tabulated 164 cases of the operation with nearly 50 per cent. cured, 25 per cent. markedly improved, and a total inclusive mortality of between 8 and 9 per cent. The cessation of catheter life has been the chief criterion of cure in these cases, and Meyer states, "that Bottini's operation may cause to disappear all the pathologic symptoms generally produced by prostatic hypertrophy, and eventually also cure a present pyelitis, is to me an established fact." As regards permanence of results, the only reliable experience is that of Bottini, who, after twenty-three years, has not seen a recurrence.

As to the indications for Bottini's operation the age of the patient and the condition of the bladder do not affect the question as they do open cutting operations, nor is pyelitis so serious a complication. The operation is equally suited to the fibro-muscular and adenomatous varieties. In those cases where the prostate is very large, soft, and bleeds easily it might be better to precede Bottini's operation by vasectomy.

It is first necessary to perform cystoscopy to observe the condition of the bladder and to detect any retro-prostatic calculus. Then again, the platinum knife must be heated to a white heat to allow for the cooling by contact with the tissues and with the fluid in the bladder. Of the several incisions, the posterior cut down to the fundus of the bladder is the most important. Meanwhile the beak of the instrument is controlled by the finger in the rectum. The elimination of scarred tissues should be left to nature; it normally occurs in from two to three weeks. It is often necessary to leave in a permanent catheter for several days.

As to the dangers of the operation, the chief are sepsis and embolism of the pulmonary artery. The risks are increased by septic nephritis, and by a large size of the prostate. Hence, early operation is indicated. In conclusion, Willy Meyer thinks that Bottini's operation bids fair to lead the van among the many operations so far devised for the radical cure of prostatic hypertrophy.

**Gonorrhœa.**—Leleneff finds that gonococci have a most destructive action on cell protoplasm, causing it to break down and vacuolate. The cocci are found not only in the mucous membranes but in the tissues, and give rise to symptoms of constitutional disorder, and to affections of any of the main systems. The nervous system suffers early, the motor, vaso-motor, and sensory nerves being alike affected. This may explain the peculiar mental state which may be observed in patients with gonorrhœa.[3]

Lyder Nicolaysen found the gonoccus in a very large proportion of cases of vulvo-vaginitis in young children, and finds that they are very susceptible to indirect infection.[4] Much attention has been devoted of late to the consideration of gonorrhœal arthritis, and the relation of gonorrhœa to chronic articular rheumatism may be more intimate than we are acquainted with. As to the treatment of gonorrhœal arthritis, McCurdy has practised and recommends aspiration and irrigation of the joint cavity with perchloride of mercury, followed by the injection of iodoform emulsion, which is allowed to remain in the joint. If the treatment be successful the pain should rapidly disappear, the temperature fall, and the general condition improve. If necessary the joint must be freely opened.[5] Besides the author's cases described, Dr. John Homans has recorded a case successfully treated by incision and drainage.[6] Protargol has been much vaunted of late by the trade and profession in the treatment of gonorrhœa. Thus Columbini sums up the results in 21 cases as excellent in every respect.[7] Others have found that the new remedies are no better than the old, and our experience as applied to protargol is the same. Certainly in some cases the drug is not only useless, but distinctly harmful.

**Syphilis.**—Adami holds that both anatomically and pathologically there is no true distinction between secondary and tertiary syphilis. Moreover, visible primary and secondary manifestations may be entirely absent, or the primary ones may be present without the others.[8]

Syphilitic phlebitis is imperfectly known. In 1881 Langenbeck removed what proved to be a gumma of the internal jugular vein. Dr. Heuzard has recently described secondary and tertiary phlebitis, the former generally affecting the saphenous veins, and recognised as a red line of congestion in their course. There is induration of the veins and œdema of the leg. Anti-syphilitic treatment is rapidly successful.[9]

With regard to the treatment of syphilis, a new and somewhat empirical remedy has been given by M. Lalande.[10] It is obtained by the prolonged action of sodium chloride upon organic matter rich in keratin—the powdered horns of a calf. He has employed it in the treatment of thirty patients, some of whom had previously been treated by mercurials. The administration is by hypodermic injection.

The Relation of Syphilis to Cancers of Mucous Membranes is the title of a suggestive paper by Dr. W. P. King (Kansas City). He himself contracted syphilis in operating upon cancerous affections of the uterus and rectum, and he gives five cases in which the two diseases seemed to be directly related. In five out of the six cases the inoculation was in the hands of doctors who had operated on cancerous uteri, and four of them died shortly afterwards.

Contrary to generally received opinions the expectancy of life in patients who have had syphilis is said to be good by Dr. J. N. Hyde.[11] If the patient has a good family history, sound constitution, good habits, and has reached a satisfactory age his expectancy of life is probably that of other individuals in similar conditions, without added risk in consequence of his specific disorder.

[1] New York Med Rec., Nov. 19, 1898. [2] New York Med. Rec., Jan. 14, 1899. [3] Epit. Brit. Med. Jour., Dec. 24, 1898. [4] Med. Rec, Nov. 26, 1898. [5] Annals of Surgery, Dec., 1898. [6] Lancet, Feb. 11, 1899, p. 392. [7] Epit. Brit. Med. Jour., Feb. 11, 1899. [8] Montreal Med. Jour., June, 1898. [9] Lancet, Oct. 15, 1898, p. 1,004. [10] Amer. Jour. of Med. Sci., Sept., 1898. [11] Med. Rec., Sept. 24, 1898.

